# Negative Correlation between Serum Cytokine Levels and Cognitive Abilities in Children with Autism Spectrum Disorder

**DOI:** 10.3390/jintelligence5020019

**Published:** 2017-05-08

**Authors:** Daimei Sasayama, Kana Kurahashi, Kayoko Oda, Takehiko Yasaki, Yoshiyuki Yamada, Nobuhiro Sugiyama, Yuji Inaba, Yuzuru Harada, Shinsuke Washizuka, Hideo Honda

**Affiliations:** 1 Mental Health Clinic for Children, Shinshu University Hospital, Matsumoto 390-8621, Japan; kanak@shinshu-u.ac.jp (K.K.); odakayo@shinshu-u.ac.jp (K.O.); hhonda@shinshu-u.ac.jp (H.H.); 2 Department of Psychiatry, Shinshu University School of Medicine, Matsumoto 390-8621, Japan; yasaki@shinshu-u.ac.jp (T.Y.), yamada0211@shinshu-u.ac.jp (Y.Y.); nsugi@shinshu-u.ac.jp (N.S.); swashi@shinshu-u.ac.jp (S.W.); 3 Department of Applied Occupational Therapy, Shinshu University School of Health Sciences, Matsumoto 390-8621, Japan; 4 Department of Pediatrics, Shinshu University School of Medicine, Matsumoto 390-8621, Japan; inabay@shinshu-u.ac.jp; 5 Nagano Prefectural Mental Wellness Center Komagane, Komagane 399-4101, Japan; yuzuru0210.mac@gmail.com

**Keywords:** autism spectrum disorder, cytokines, interleukin-6, interferon-γ, intelligence

## Abstract

Evidence suggests that cytokines may be one of the major factors influencing cognitive development in those with autism spectrum disorder (ASD). To shed light on the neural and cognitive mechanisms of ASD, we investigated the association between peripheral cytokine levels and cognitive profiles in children with ASD. The serum levels of 10 cytokines (granulocyte macrophage colony-stimulating factor, interferon (IFN)-γ, interleukin (IL)-1β, IL-2, IL-4, IL-5, IL-6, IL-8, IL-10, and tumor necrosis factor-α) were examined in 14 children with ASD using the Human Ultrasensitive Cytokine Magnetic 10-Plex Panel for the Luminex platform. The Wechsler Intelligence Scale for Children (WISC) was administered to each subject, and the relationships between WISC scores and serum levels of the cytokines were examined. The full-scale intelligence quotient (IQ) was significantly negatively correlated with the levels of IL-6 (Spearman’s rank, *p* < 0.0001, false discovery rate *q* < 0.01). The levels of IL-6 and IFN-γ showed significant negative correlations with the verbal comprehension index (*p* < 0.001, *q* < 0.01) and working memory index (*p* < 0.01, *q* < 0.05), respectively. No other cytokines were significantly correlated with full-scale IQ or with any of the subscale scores of the WISC. The present results suggest negative correlations of IL-6 and IFN-γ levels with cognitive development of children with ASD. Our preliminary findings add to the evidence that cytokines may play a role in the neural development in ASD.

## 1. Introduction

Several lines of evidence suggest that proinflammatory cytokines have influence on neural development and function [1,2]. Previous studies have shown that higher peripheral levels of interleukin-6 (IL-6) are associated with cognitive decline in healthy subjects [3,4] and in those with Alzheimer’s disease [5]. Studies that examined the association between cognitive performance and genetic polymorphisms of cytokine genes also suggest the involvement of cytokines in altering cognitive performance [6,7]. Animal studies have also shown that IL-6 is involved in cognitive functioning. A study using transgenic mice showed that IL-6 deletion had a facilitatory effect on learning and memory [8]. Furthermore, Heyser et al. [9] showed that chronic IL-6 expression from astrocytes in the brain can induce decline in avoidance learning performance. Taken together, the persistence of excessive signaling of proinflammatory cytokines may have a negative impact on the cognitive performance. 

The precise mechanism of how cytokines exert influence on cognitive ability remains unknown; however, a number of mechanisms are likely to be involved. Interference of normal neural development caused by cytokine dysregulation, as reviewed by Deverman and Patterson [10], may be one of the mechanisms of how cytokines affect cognitive ability. Indeed, a number of studies have shown that cytokines may be involved in the pathogenesis of disorders associated with abnormal neural development, such as schizophrenia [11,12], autism [13,14], and other neurodevelopmental disorders [15,16]. 

Autism spectrum disorder (ASD) is a group of neurodevelopmental disorders characterized by atypical social interactions, impaired communication, and idiosyncratic, repetitive, or restrictive behaviors. A birth cohort study reported that 1% to 2% of the population is diagnosed as ASD by seven years of age [17]. Although the etiology of ASD remains uncertain, evidence to date indicates the involvement of the immune system in the pathogenesis of ASD [18]. A number of studies have shown altered peripheral cytokine levels in subjects with ASD [19,20]. Individuals with ASD often present with uneven cognitive profiles [21]. A previous study showed that intelligence quotient (IQ) discrepancy between verbal and performance IQ was correlated with some of the core features of autism [22]. We hypothesized that cytokines may be one of the major factors influencing cognitive ability in those with ASD. To shed light on the neural and cognitive mechanisms of ASD, we examined the association between peripheral cytokine levels and cognitive profiles in children with ASD.

## 2. Materials and Methods 

### 2.1. Subjects

Subjects were 14 children diagnosed with ASD or pervasive developmental disorder according to Diagnostic and Statistical Manual of Mental Disorders (DSM), 5th edition or DSM, 4th edition, respectively, by an experienced child psychiatrist. All subjects were biologically unrelated Japanese individuals. Participants were excluded if they had a prior medical history of central nervous system disease or severe head injury, if they met the DSM-IV criteria for substance abuse, substance dependence, or mental retardation, or if they suffered from any inflammatory, infectious, or systemic immune diseases at the time of assessment. The study protocol was approved by the ethics committee at the Shinshu University School of Medicine, Japan. Verbal consent was obtained from all participants and written informed consent from their parents after description of the study.

### 2.2. Assessment of Cognitive Functioning

All participants were administered the Japanese version of either the Wechsler Intelligence Scale for Children, 3rd edition [23] or the WISC, 4th edition [24] by a clinical psychologist. Since the version of the WISC used at the Shinshu University Hospital, where the study took place, was updated during the study period, the WISC-III was administered to participants before September 2013, and the WISC-IV was administered thereafter.

### 2.3. Cytokine Measurements

Serum samples were collected in serum separating tubes and were allowed to clot for 1 h at room temperature. The samples were centrifuged for 10 min at 4 °C and were aliquoted and stored at −80 °C until they were assayed. Serum levels of granulocyte macrophage colony-stimulating factor (GM-CSF), interferon (IFN)-γ, IL-1β, IL-2, IL-4, IL-5, IL-6, IL-8, IL-10, and tumor necrosis factor (TNF)-α were quantified by Human Ultrasensitive Cytokine Magnetic 10-Plex Panel for the Luminex^®^ platform (Life Technologies, Carlsbad, CA, USA) according to the manufacturer’s instructions. Each sample measurement was performed in duplicate, and all samples were run on the same assay. Cytokine measurements below the lower limit of detection as determined by the standard curve were assigned a value of the limit of detection. The lower limit of detection for each cytokine was as follows: GM-CSF (0.528 pg/mL), IFN-γ (0.204 pg/mL), IL-1β (0.286 pg/mL), IL-2 (0.376 pg/mL), IL-4 (0.574 pg/mL), IL-5 (0.551 pg/mL), IL-6 (0.178 pg/mL), IL-8 (0.494 pg/mL), IL-10 (0.665 pg/mL), and TNF-α (0.304 pg/mL). Cytokines whose detection rates were 50% or less were excluded from the analyses.

### 2.4. Statistical Analysis

Differences between groups were compared using the Mann–Whitney U test. Statistical tests were two tailed and statistical significance was considered when *p* < 0.05. Correlations between cytokine levels and basic clinical data were examined by Spearman’s correlation analysis. Correlations of cytokine levels with the full-scale IQ (FSIQ) and subscale scores of the WISC (i.e., Verbal Comprehension Index (VCI), Perceptual Reasoning Index (PRI) or Perceptual Organization Index (POI), Working Memory Index (WMI) or Freedom and Distractibility Index (FDI), and Processing Speed Index (PSI)) were analyzed by using Spearman’s partial rank correlation test controlling for the version of the WISC administered (WISC-III or WISC-IV). A false discovery rate (FDR) *q* value was used to correct for multiple comparisons (i.e., 8 cytokines included in the analyses × 5 scores = 40 tests). FDR *q* values were calculated based on the Benjamini and Hochberg method. Statistical significance was considered when *q* < 0.05. All statistical analyses were performed using R software version 3.2.3 [25].

## 3. Results

The clinical characteristics of the participants and the WISC scores are presented in Table 1. No significant difference in full-scale IQ (FSIQ) or any of the subscale scores (i.e., VCI, PRI or POI, WMI or FDI, and PSI) was observed between those who were administered WISC-III and those administered WISC-IV. 

The detection rates of the cytokines were as follows: GM-CSF (0%), IFN-γ (50%), IL-1β (50%), IL-2 (71%), IL-4 (50%), IL-5 (21%), IL-6 (79%), IL-8 (100%), IL-10 (71%), and TNF-α (50%). Only cytokines with detection rates 50% or above, i.e., IFN-γ, IL-1β, IL-2, IL-4, IL-6, IL-8, IL-10, and TNF-α, were included in further analyses. The intra-assay coefficients of variation were as follows: IFN-γ (12.8%), IL-1β (7.5%), IL-2 (11.7%), IL-4 (10.8%), IL-6 (9.9%), IL-8 (6.7%), IL-10 (12.4%), and TNF-α (6.44%).

**Figure 1:**
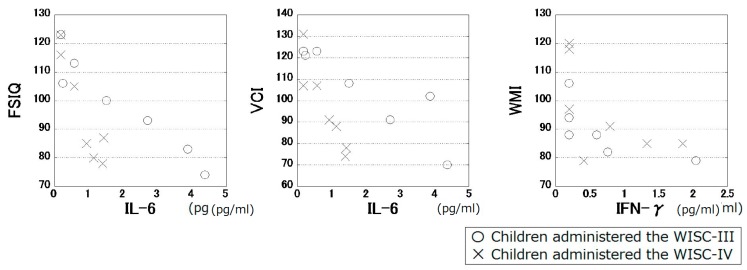
Significant correlations of serum IL-6 levels with FSIQ and VCI were observed in children administered the WISC-III as well as in those administered the WISC-IV.

Body weight was significantly positively correlated with the levels of IL-10 (ρ = 0.55, *p* = 0.041) and TNF-α (ρ = 0.57, *p* = 0.035). No significant effect of sex, age, or medication status on cytokine levels was observed. The serum level of IL-6 was significantly negatively correlated with FSIQ (ρ = −0.89, *p* = 0.000050, *q* = 0.0020) as well as with VCI (ρ = −0.85, *p* = 0.00021, *q* = 0.0042). The serum level of IFN-γ was significantly negatively correlated with WMI (ρ = −0.77, *p* = 0.0021, *q* = 0.027). No other significant correlations were observed between cytokine levels and the WISC scores. Figure 1 shows the significant associations found between cytokines and the WISC scores.

## 4. Discussion

The present study showed significant negative correlations between cytokine levels and cognitive abilities in children with ASD. Our results suggest that higher levels of cytokines in children with ASD may have negative impact on cognitive function. 

Several studies that examined association between genetic variations and intelligence suggest that cytokines play a role in cognitive functioning. It has been reported that genetic variations in the gene encoding IL-1β, which stimulates IL-6 production [26], may be associated with cognitive performance [6,27,28]. Furthermore, we have previously shown that the Ala allele of IL6R Asp358Ala polymorphism, which is associated with higher circulating IL-6 and soluble IL-6R levels [12], was associated with lower verbal IQ in healthy adults [7]. These findings are in line with the present findings that excessive IL-6 signaling may impair verbal cognitive ability. 

The present findings were also consistent with previous studies that showed an association between high peripheral levels of IL-6 and cognitive decline in healthy subjects [3,4]. A few other studies, however, did not find a significant association between peripheral IL-6 levels and cognitive ability in elderly subjects [29,30]. The inconsistent findings may, in part, be due to differences in subject selection. Because numerous factors contribute to cognitive ability, only a weak association may be detected in a heterogeneous group of subjects between the intelligence scores and a particular cause of cognitive impairment. We speculate that one of the reasons for the relatively strong correlation observed between IL-6 level and cognitive ability in the present study may be because we focused on cognitive profiles in only children with ASD. The present results suggest that among various factors that impair the development of verbal cognitive ability, excessive IL-6 may have a particularly high degree of influence in children with ASD.

Some studies also suggest the involvement of IFN-γ in cognitive impairment. Schrier et al. [31] reported that the presence of IFN-γ expressing CD8+ T cells contribute to increased risk of neurocognitive disorder in patients with HIV infection. Jones et al. [32] reported that mothers of children with ASD comorbid with intellectual disability showed significantly elevated serum cytokines including IFN-γ and IL-6 at mid-gestational stage. On the other hand, increase in circulating levels of IFN-γ at birth was shown to be related to lower odds of developing low performance IQ [33]. Furthermore, studies in mice showed that IFN-γ enhances neurogenesis [34,35] and improves spatial learning and memory performance [35]. These contradicting findings infer that the observed associations could be caused indirectly as a part of a complex bidirectional network between cytokines and the central nervous system.

The major limitations of the present study were the small sample size and the cross-sectional design of the study, which does not allow causal interpretations of the data. Future studies are necessary to confirm our findings by prospectively examining the longitudinal effects of the peripheral cytokine levels in a larger group of children with ASD. Despite these limitations, the present findings suggest an involvement of proinflammatory cytokines in cognitive development of children with ASD. Our preliminary findings add to the evidence that cytokines may play a role in the neural development in ASD.

## Figures and Tables

**Table 1:** Clinical characteristics, Wechsler Intelligence Scale for Children (WISC) scores, and cytokine levels.

Clinical Characteristics	Subjects Administered the WISC-III	Subjects Administered the WISC-IV	Total
N (boys/girls)	7 (5/2)	7 (4/3)	14 (9/5)
Age (years)	11.7 ± 1.7	11.6 ± 2.6	11.6 ± 2.1
Body Weight (kg)	45.6 ± 8.8	54.4 ± 26.2	50.0 ± 19.3
Medication status			
On any mediation	4	3	7
Stimulant	2	2	4
Atomoxetine	1	0	1
Guanfacine	0	1	1
Atypical antipsychotics	1	0	1
WISC scores (standard scores)			
FSIQ	98.9 ± 17.0	96.3 ± 18.2	97.6 ± 17.0
VCI	105.4 ± 19.8	96.6 ± 19.8	101.0 ± 19.6
POI (WISC-III) or PRI (WISC-IV)	96.7 ± 13.5	96.0 ± 20.1	96.4 ± 16.4
FDI (WISC-III) or WMI (WISC-IV)	89.3 ± 8.8	96.4 ± 16.4	92.9 ± 13.2
PSI	94.6 ± 19.8	96.9 ± 13.3	95.7 ± 16.2
Time of blood collection	10.34 h ± 158 min	13.11 h ± 169 min	11.53 h ± 177 min
IFN-γ (pg/mL)	0.6 ± 0.7	0.7 ± 0.7	0.7 ± 0.6
IL-1β (pg/mL)	0.5 ± 0.3	0.5 ± 0.3	0.5 ± 0.3
IL-2 (pg/mL)	5.4 ± 8.7	2.0 ± 1.7	3.7 ± 6.3
IL-4 (pg/mL)	2.2 ± 2.8	2.1 ± 1.7	2.1 ± 2.2
IL-6 (pg/mL)	1.9 ± 1.7	0.8 ± 0.5	1.4 ± 1.4
IL-8 (pg/mL)	10.2 ± 10.3	7.8 ± 6.4	9.0 ± 8.3
IL-10 (pg/mL)	1.4 ± 0.9	1.8 ± 1.5	1.6 ± 1.2
TNF-α (pg/mL)	0.6 ± 0.6	1.1 ± 0.8	0.8 ± 0.7
